# Mental regulation of students’ psychological states

**DOI:** 10.1192/j.eurpsy.2022.1608

**Published:** 2022-09-01

**Authors:** M. Kartasheva, A. Prokhorov, A. Chernov, M. Yusupov

**Affiliations:** Kazan Federal University, Institute Of Psychology And Education, Kazan, Russian Federation

**Keywords:** psychological state, self-regulation, academic activity

## Abstract

**Introduction:**

The main scientific task of the study is at the intersection of two fundamental areas of psychology - self-regulation of human mental states and management of educational activities. In the context of these problems, we study the self-regulation of states in academic activities.

**Objectives:**

The purpose of the study is to show the patterns of mental organization of the person that ensure the regulation of states.

**Methods:**

The theoretical basis of the study is the system approach. The typical methods and techniques of self-regulation of states, as well as the influence of mental structures on students’ self-regulation have been studied. To solve the problems, standardized psychological tests have been used (23 methods, 303 indicators). The research involved 206 students.

**Results:**

We have revealed the features of the relationship between the quality of students’ subject training, regulatory abilities and mental states. It indicates that the effectiveness of students’ mental states self-regulation significantly affects the productivity of the semester exam. It has been established the properties of the personality providing high efficiency of self-regulation (adequacy, awareness, independence and assertiveness). Students with high self-regulation efficiency use a wide range of regulatory means.

**Conclusions:**

The study of the influence of the mental structures on self-regulation and regulatory abilities of students confirmed the hypothesis that characteristics of mental organization play the central role in the regulation of psychological states. The research has been carried out with the financial support of the RFBR, project No. 19-29-07072.

**Disclosure:**

No significant relationships.

